# Rab11a in the spinal cord: an essential contributor to complete Freund’s adjuvant-induced inflammatory pain in mice

**DOI:** 10.1186/s13041-023-01057-3

**Published:** 2023-09-28

**Authors:** Jun-Xiang Gu, Jian Wang, Fu-Juan Ma, Miao-Miao Liu, Si-Hai Chen, Yi Wei, Yi-Fan Xiao, Pei-Yuan Lv, Xin Liu, Jian-Qiang Qu, Xian-Xia Yan, Tao Chen

**Affiliations:** 1 Department of Neurosurgery, the Second Affiliated Hospital of Xi’an Jiaotong University, Xi’an, China; 2 Department of Anatomy and K.K. Leung Brain Research Centre, Fourth Military Medical University, Xi’an, China; 3 Department of Neurosurgery, Tangdu Hospital, Fourth Military Medical University, Xi’an, China; 4 School of Medicine, Northwest University, Xi’an, China; 5 Department of Respiratory and Critical Care Medicine, Tangdu Hospital, Fourth Military Medical University, Xi’an, China; 6 Department of Psychiatry, Xiaogan Mental Health Center, Xiaogan, China; 7 Department of Stomatology, The 960th Hospital of People’s Liberation Army, Jinan, Shandong China

**Keywords:** Rab11a, Spinal dorsal horn, Inflammatory pain, NMDA receptor

## Abstract

Inflammatory pain is a commonly observed clinical symptom in a range of acute and chronic diseases. However, the mechanism of inflammatory pain is far from clear yet. Rab11a, a small molecule guanosine triphosphate enzyme, is reported to regulate orofacial inflammatory pain in our previous works. However, the mechanism of Rab11a’s involvement in the regulation of inflammatory pain remains obscure. Here, we aim to elucidate the potential mechanisms through which Rab11a contributes to the development of inflammatory pain in the spinal level. It’s shown that neurons, rather than glial cells, were the primary cell type expressing Rab11a in the spinal dorsal horn (SDH). After intra-plantar injection of CFA, both the number of Fos/Rab11a-immunopositive neurons and the expression of Rab11a were increased. Administration of Rab11a-shRNA into the SDH resulted in significantly analgesic effect in mice with CFA injection. Application of Rab11a-shRNA also reduced the NMDA receptor-mediated excitatory post-synaptic current (EPSC) and the spike number of neurons in lamina II of the SDH in mice with CFA injection, without affecting the presynaptic glutamate release and the postsynaptic AMPA receptor-mediated EPSC. Our results thus suggest that the enhanced expression of neuronal Rab11a may be important for the process of inflammatory pain in mice with CFA injection, which is likely mediated by Rab11a’s potentiation of the competence of post-synaptic NMDAR and spiking of SDH neurons.

## Introduction

Inflammatory pain widely exists in varieties of acute and chronic diseases which are caused through infectious disease, tissue damage, persistent inflammation, and so on [1, 2]. Previous studies [3–5] indicate that sensory neurons in the spinal dorsal horn (SDH) play a key role in the transmission of peripheral pain information to the central nervous system. The SDH is the key area of central nervous system that receives the central terminals of primary afferent neurons and descending supraspinal modulatory projections [6, 7]. Therefore, it is particularly critical to explore the mechanism of the formation and development of inflammatory pain in the SDH.

Rab11a, a member of small molecule guanosine triphosphate (GTP) enzymes of Rab family [8–10], is reported to be involved in traumatic brain injury, Alzheimer’s disease, and so on [11–13]. Our recent study reveals that Rab11a is closely related to Complete Freund’s Adjuvant (CFA)-induced orofacial pain [14]. Downregulation of Rab11a expression in the caudal part of the spinal trigeminal nucleus (Sp5C) significantly alleviates CFA-induced orofacial mechanical and thermal pain. However, how is Rab11a involved in the development and worsening of inflammatory pain remains obscure yet.

In the present study, the critical role and potential mechanism of Rab11a in the SDH were investigated after hind paw CFA intra-planter injection. Our results showed that Rab11a immunoactivities were mainly enriched in SDH neurons. After CFA injection, the expression of Rab11a and the activities of Rab11a-containing SDH neurons were enhanced. Application of Rab11a-shRNA reduced the CFA injection induced mechanical allodynia, which is likely mediated by Rab11a-shRNA’s inhibition of the NMDA receptor (NMDAR)-mediated excitatory postsynaptic currents (EPSC), as well as the neuronal spiking in the SDH. Our results thus suggest that Rab11a is an essential contributor to CFA-induced inflammatory pain in mice and Rab11a may be a potential therapeutic target for pain treatment.

## Results

### Rab11a positive cells are concentrated in neurons in the SDH

**Fig. 1:**
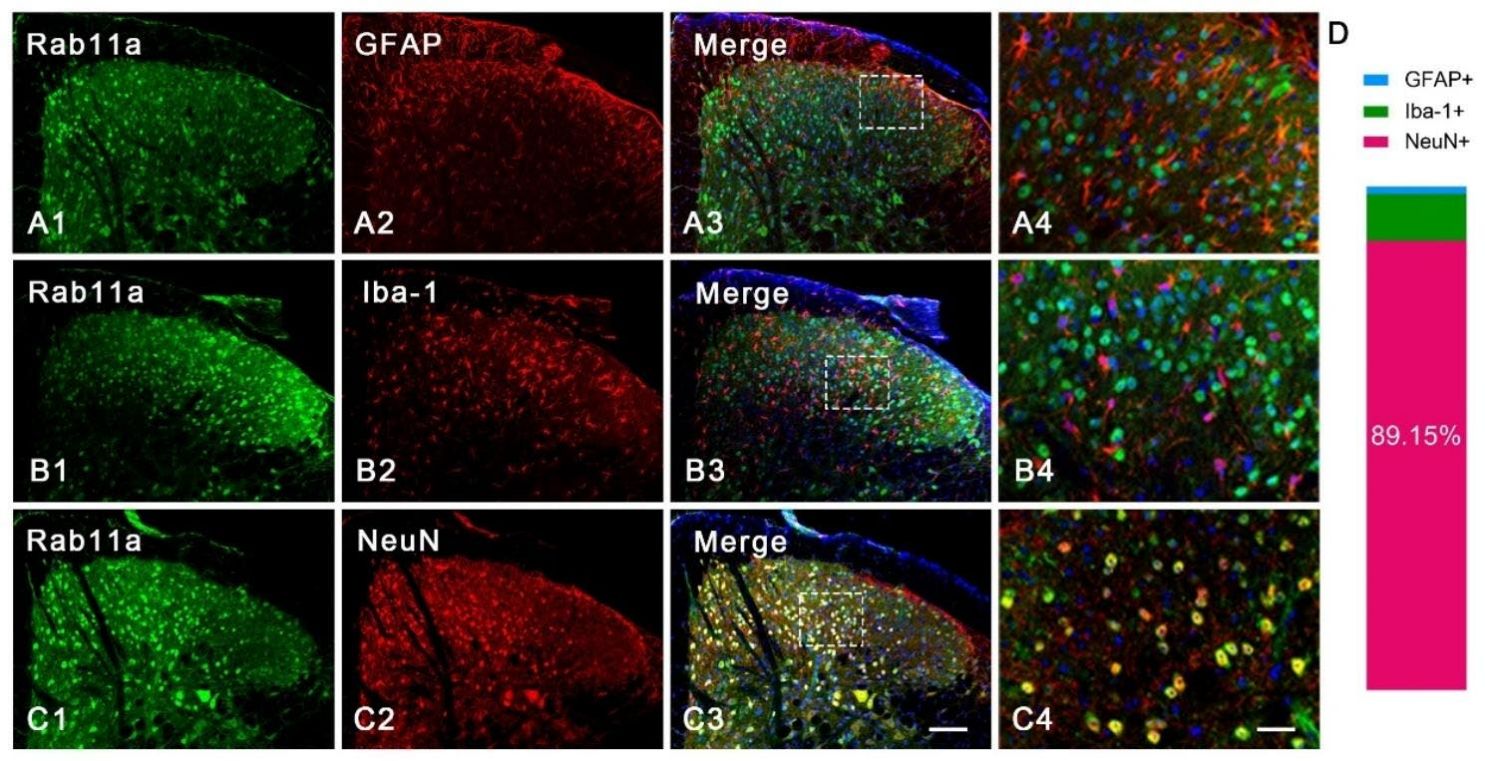
**The expression of Rab11a in the SDH.** **A1**, **B1**, **C1**, the expression of Rab11a-immunoreactivities in the SDH. **A2**, **B2**, **C2**, the expression of GFAP- (**A2**), Iba1- (**B2**) and NeuN-immunoreactivities (**C2**) in the SDH. **A3**, **B3**, **C3**, the merged figures from **A1**-**A2** (**A4**), **B1-B2 (B4)** and **C1-C2 (C4)** respectively, which are counter-stained with DAPI. The entangled areas in **A3-C3** are enlarged and shown as **A4-C4**. **D**, the ratio of Rab11a in GFAP (1.59%)-, IBa1 (9.26%)- and NeuN (89.15%)-immunoreactive cells (n = 3 mice in each group). Bars equal to100 µm in (**A1-C3**) and 50 μm in (**A4-C4**).

The Rab11a positive cells in the SDH were checked by immunofluorescence staining. It’s shown that 89.15% Rab11a-immunoreactivities were in cytoplasm of neurons (Fig. 1C1-C4 and D), which is consistent with previous reports [14]. In contrast, only a small proportion of Rab11a-positive cells were co-labeled with glial fibrillary acidic protein (GFAP) (Fig. 1A1-A4) and Ionized calcium-binding adapter molecule 1 (Iba-1) (Fig. 1B1-B4). These results indicate that Rab11a is mainly expressed in neuron, but not astrocyte and microglia, in the SDH.

### Rab11a expression level is significantly correlated with the process of inflammatory pain

**Fig. 2:**
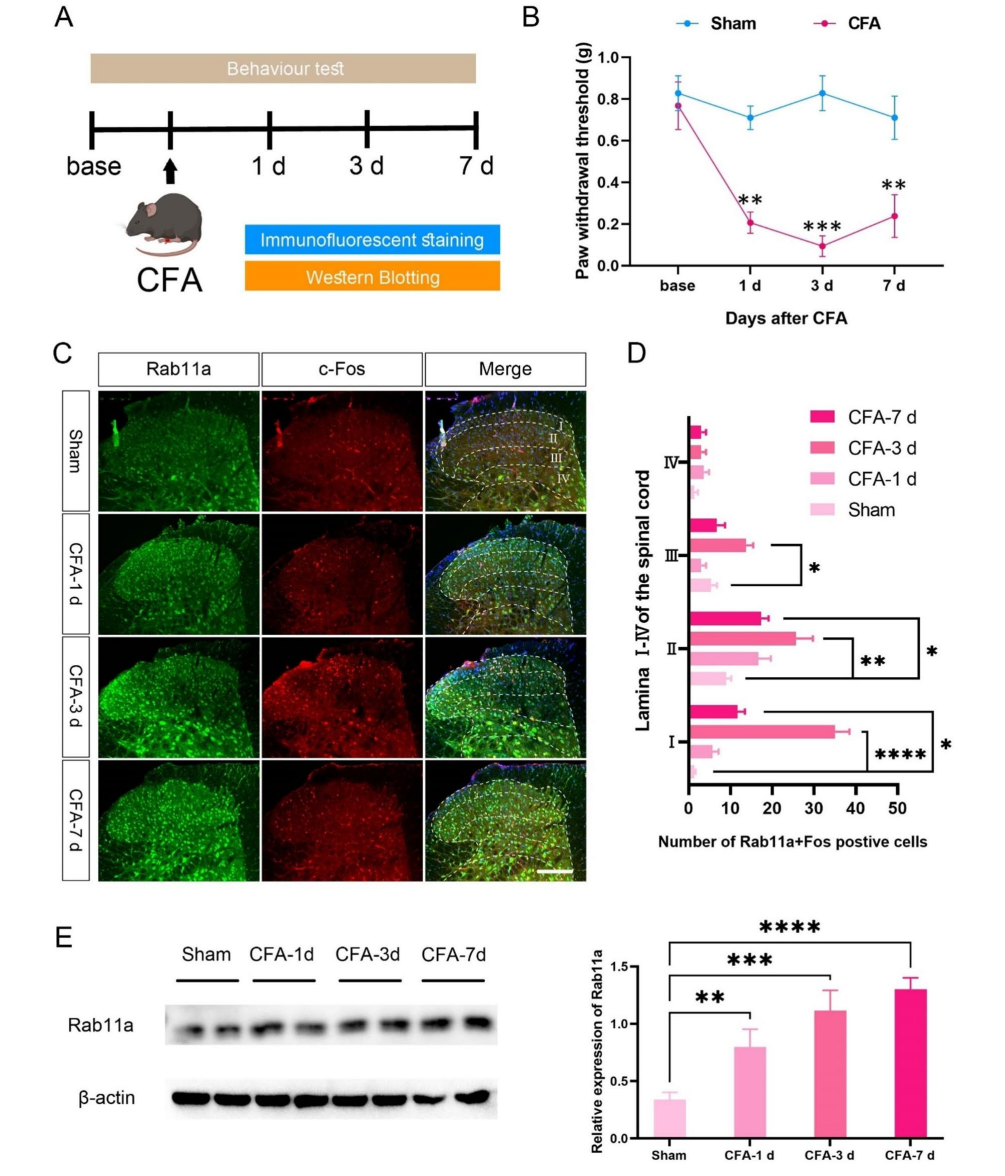
**Rab11a expression level is correlated with the process of inflammatory pain** **A**, the experimental procedure. **B**, the PWMT in sham and CFA groups (n = 6 mice in each group, *Two-Way ANOVA* followed by *Tukey’s post-hoc* test, *F*_*(1,4)*_ = 139.5, *P* = 0.0003). **C**, immunofluorescent samples showing the Rab11a positive cells expressing Fos protein in lamina I-IV of the spinal cord after CFA injection. **D**, the number of Rab11a-Fos positive cells in lamina I-IV (n = 3 mice in each group, *One-Way ANOVA* followed by *Tukey’s post-hoc* test, I: *F*_*(3,8)*_ = 51.92, *P* < 0.0001; II: *F*_*(3,8)*_ = 6.257, *P* = 0.0171; III: *F*_*(3,8)*_ = 7.903, *P* = 0.0089). **E**, samples and quantitative analysis of Rab11a expression levels via western blotting (n = 3 mice in each group, *One-Way ANOVA* followed by *Tukey’s post-hoc* test, *F*_*(3,8)*_ = 30.60, *P* < 0.0001). *P < 0.05, **P < 0.01, ***P < 0.001, ****P < 0.0001. Bars equal to100 µm in C

We then tested whether the expression of Rab11a is correlated with pain process (Fig. 2A). After intra-plantar injection of CFA, the paw withdrawal mechanical threshold (PWMT) of the ipsilateral hind paw was significantly decreased from 1st to 7th day post injection (Fig. 2B). Meanwhile, the number of Rab11a-positive cells co-expressing Fos protein in lamina I-III increased parallel with CFA-induced chronic inflammatory pain (Fig. 2 C-2D). In consistence, western blotting analysis revealed a sequential upregulation of Rab11a expression level on the 1st, 3rd, and 7th day post-CFA injection (Fig. 2E).

### Knockdown of Rab11a reverses CFA-induced allodynia

**Fig. 3:**
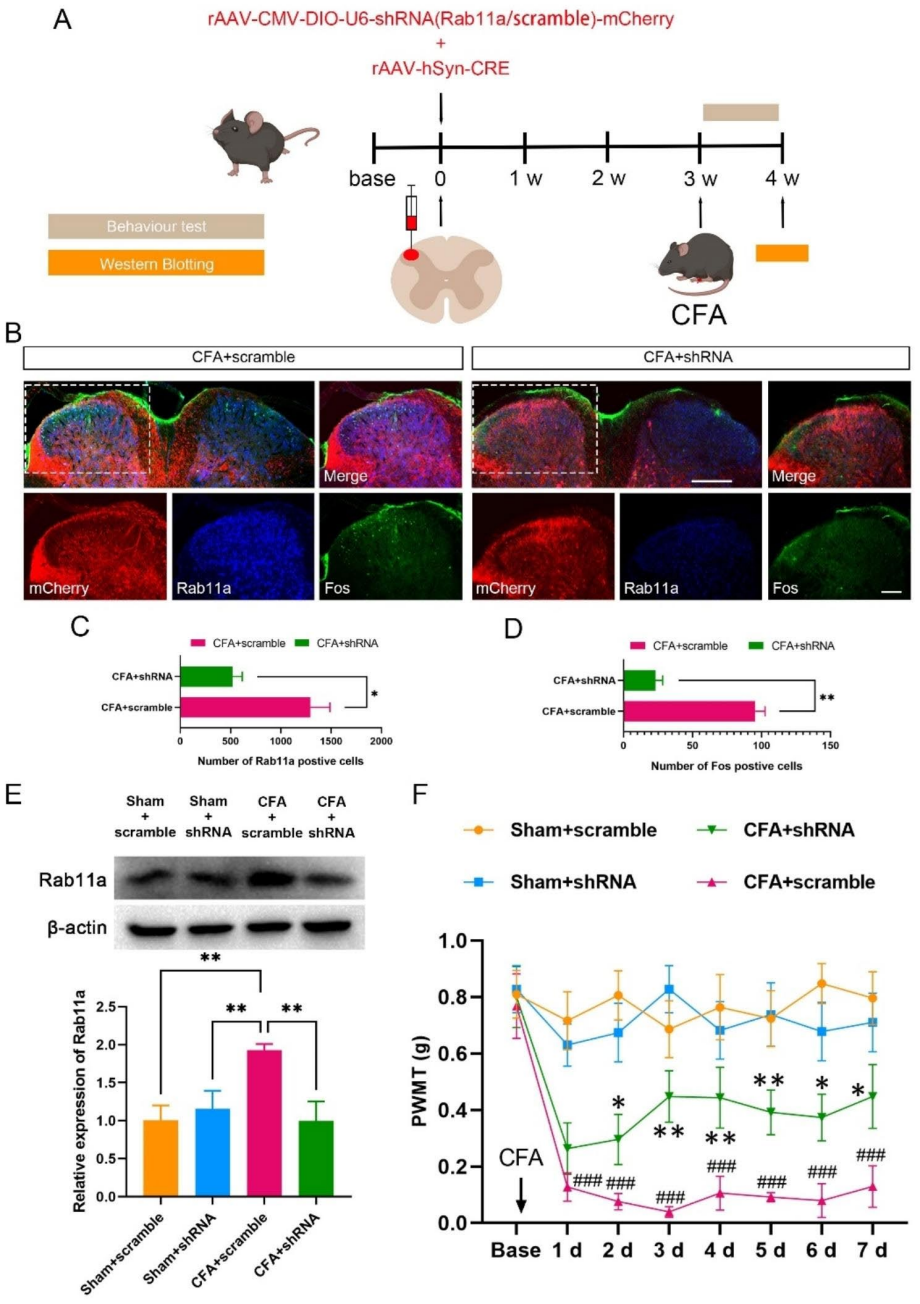
**Rab11a-shRNA reverses CFA induced allodynia** **A**, Experimental procedure of Rab11a-shRNA application. **B**, immunofluorescent samples showing that Rab11a-shRNA reversed the increased number of Rab11a positive cells (**C**, n = 3 mice in each group, unpaired *t*-test, *t* = 3.558, *P* = 0.0236) and Fos positive cells (**D**, n = 3 mice in each group, unpaired *t*-test, *t* = 8.082, *P* = 0.0013) induced via CFA injection. **E**, Expression level of Rab11a in sham + scrambled-shRNA (sham + scramble), sham + Rab11a-shRNA (sham + shRNA), CFA + scramble, and CFA + shRNA groups. CFA injection increased Rab11a expression, which was significantly reversed by Rab11a-shRNA (n = 3 mice in each group, *One-Way ANOVA* followed by *Tukey’s post-hoc* test, *F*_*(3,8)*_ = 14.27, *P* = 0.0014, **P < 0.01). **F**, PWMT of sham + scramble, CFA + scramble, sham + shRNA and CFA + shRNA groups. CFA injection reduced the PWMT, which was reversed by Rab11a-shRNA (n = 6 mice in each group, *Two-Way ANOVA* followed by *Tukey’s post-hoc* test, *F*_*(3,16)*_ = 57.22, *P* < 0.0001). Sham + scramble vs. CFA + scramble, ^###^*P* < 0.001; sham + shRNA vs. CFA + shRNA, *P < 0.05, **P < 0.01. Bars equal to200 µm and 100 μm in B

The morphological results suggest that the expression of Rab11a is correlated with the process of inflammatory pain, we thus want to test whether knockdown of the Rab11a will rescue the pain responses. Rab11a-specific short hairpin RNA (Rab11a-shRNA) binding to AAV2/9 virus or control virus (scrambled-shRNA), which has been confirmed in previous work [14, 15], was then injected into the SDH ipsilateral to the CFA injected paw (Fig. 3A). Immunofluorescence staining results showed that Rab11a-shRNA could specifically knock down the expression of Rab11a and reduce the number of Fos-positive cells in the virus injection area (Fig. 3B-D). Western blotting results revealed a significant increase of Rab11a expression level in the SDH of the CFA + scrambled-shRNA (CFA + scramble) group compared to the sham + scrambled-shRNA (Sham + scramble) group, which was significantly reduced by administration of Rab11a-shRNA (Fig. 3E). Behaviorally, administration of Rab11a-shRNA significantly increased the PWMT of the mice with CFA injection in comparison to the CFA + scramble group (Fig. 3F), indicating the potent analgesic effect with knockdown of Rab11a.

### Rab11a-shRNA decreases the amplitude of NMDA receptor-mediated EPSC

**Fig. 4:**
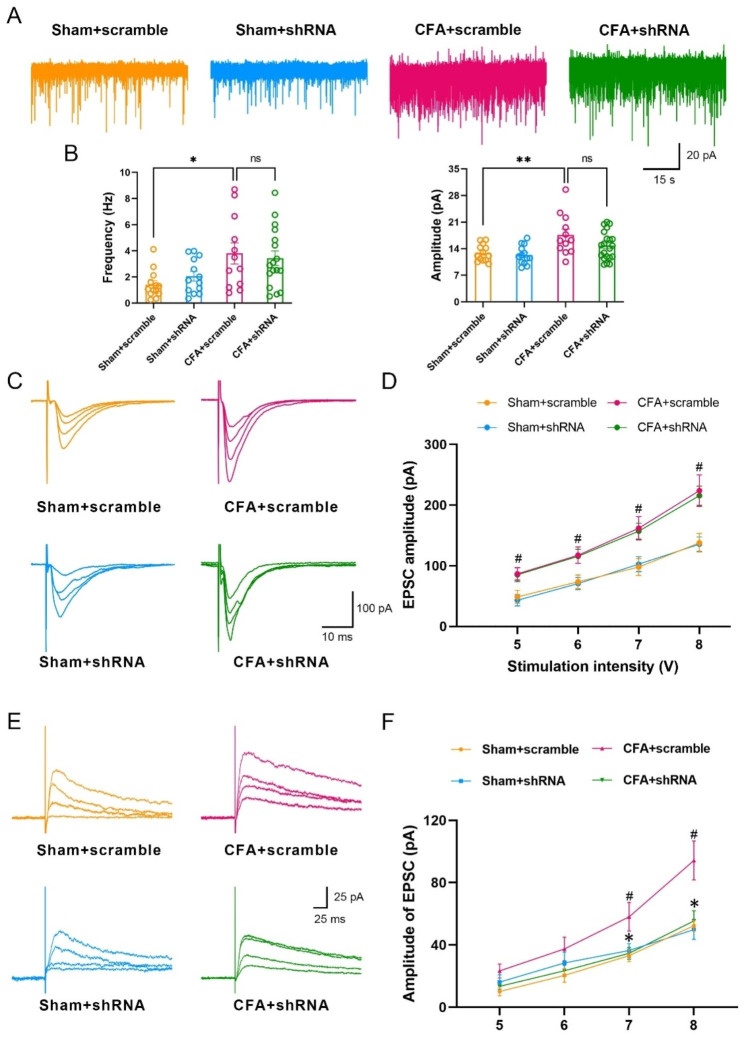
**Rab11a-shRNA decreases the amplitude of NMDA receptor-mediated EPSC.** **A**, Representative trace of sEPSC in Sham + scrambled-shRNA (Sham + scramble, n = 13 cells of 3 mice), Sham + Rab11a-shRNA (Sham + shRNA, n = 13 cells of 3 mice), CFA + scrambled-shRNA (CFA + scramble, n = 12 cells of 3 mice), and CFA + Rab11a-shRNA (CFA + shRNA, n = 17 cells of 4 mice) groups. **B**, The frequency (*One-Way ANOVA* followed by *Tukey’s post-hoc* test, *F*_*(3,51)*_ = 4.240, *P* = 0.0095, *P < 0.05) and amplitude (*One-Way ANOVA* followed by *Tukey’s post-hoc* test, *F*_*(3,55)*_ = 5.411, *P* = 0.0025, **P < 0.01) of sEPSC. **C**, Representative traces of AMPA receptor mediated EPSC from Sham + scramble, Sham + shRNA, CFA + scramble, and CFA + shRNA groups. **D**, Averaged amplitude of AMPAR mediated EPSC from sham + scramble (n = 9 cells of 3 mice), sham + shRNA (n = 9 cells of 3 mice), CFA + scramble (n = 8 cells of 3 mice) and CFA + shRNA (n = 8 cells of 3 mice) groups (*Two-Way ANOVA* followed by *Tukey’s post-hoc* test, *F*_*(3,30)*_ = 6.232, *P* = 0.0020). CFA increased the amplitude of NMDA receptor mediated EPSC when voltage of 5 V (*P* = 0.0294), 6 V (*P* = 0.0447), 7 V (*P* = 0.0494) and 8 V (*P* = 0.0143) were applied. However, the shRNA injection group did not produce a significantly lower amplitude compared with the CFA + scramble group. **E**, Representative traces of NMDA receptor mediated EPSC from Sham + scramble, Sham + shRNA, CFA + scramble, and CFA + shRNA groups. **F**, Averaged amplitude of NMDAR mediated EPSC from sham + scramble (n = 8 cells of 3 mice), sham + shRNA (n = 9 cells of 3 mice), CFA + scramble (n = 9 cells of 3 mice) and CFA + shRNA (n = 10 cells of 3 mice) groups (*Two-Way ANOVA* followed by *Tukey’s post-hoc* test, *F*_*(3,32)*_ = 4.901, *P* = 0.0065, *P < 0.05). CFA increased the amplitude of NMDA receptor mediated EPSC when voltage of 7 V (*P* = 0.0409) and 8 V (*P* = 0.0284) were applied, and the shRNA injection group produced a significantly lower amplitude in response to voltage of 7 V (*P* = 0.0417) and 8 V (*P* < 0.0310) compared with the CFA + scramble group

We then wanted to know how Rab11a is involved in the synaptic or neuronal excitability in the SDH. Neurons in lamina II of the SDH were recorded by using whole-cell patch-clamp recordings method. We found that, with incubation of scrambled-shRNA, the frequency and amplitude of spontaneous excitatory postsynaptic currents (sEPSC) were increased in the CFA group compared to the sham group. However, neither the frequency nor the amplitude of sEPSC was affected with incubation of Rab11a-shRNA (Fig. 4A-B). These results indicate that knockdown of Rab11a does not affect the presynaptic glutamate release and AMPA receptor mediated postsynaptic responses. Therefore, we recorded the input (stimulation intensity)-output (amplitude) curve (I-O curve) of AMPAR-EPSCs in lamina II neurons of the SDH and found that the slope of the I-O curve was steeper in mice of CFA-scramble group, compared with Sham-scramble group. However, the slope of the I-O curve did not change in CFA-shRNA group, compared with CFA-scramble group (Fig. 4 C-D). It was further observed that the amplitude of NMDA receptor-mediated EPSC was potentiated in CFA group, which was reduced by incubation of Rab11a-shRNA (Fig. 4E-F), suggesting that Rab11a is involved in the enhanced NMDAR-mediated post-synaptic responses after CFA injection.

### Rab11a-shRNA decreases CFA-induced hyperexcitation of SDH neurons

**Fig. 5:**
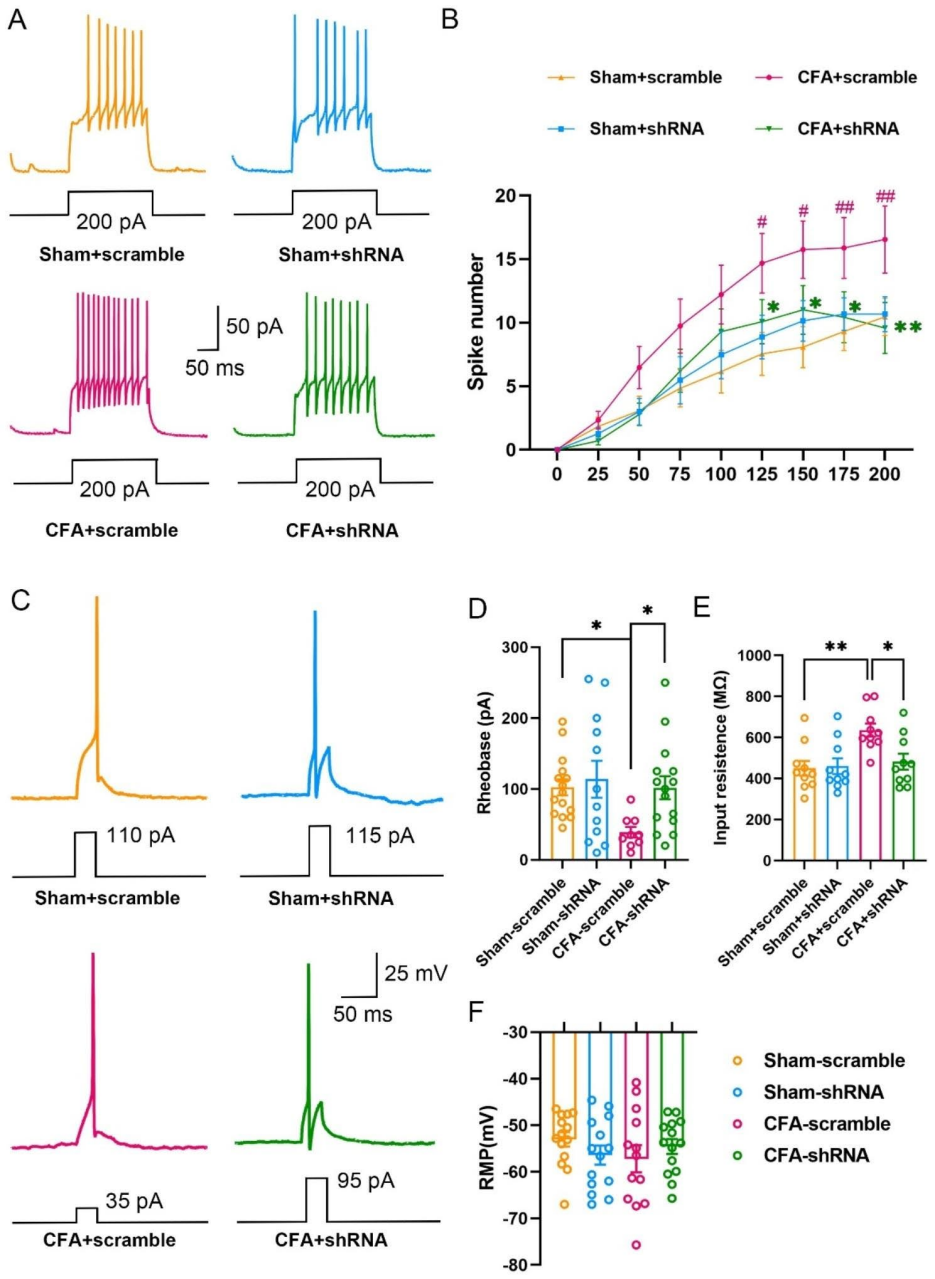
**Rab11a-shRNA decreases the excitation of SDH neurons** **A**, Representative traces of spike numbers in sham + scrambled-shRNA (sham + scramble), sham + Rab11a-shRNA (sham + shRNA), CFA + scrambled-shRNA (CFA + scramble), and CFA + Rab11a-shRNA (CFA + shRNA) groups. **B**, Averaged spike numbers induced by step currents injection (300 ms, 0-200 pA) (Two way ANOVA followed by Turkey post hoc test, F = P=, vs. ** P; vs. ^###^ P). **C**, Representative traces of the first AP of neurons in SDH induced by + 5 pA step depolarizing current intracellular injections. **D-F**, the rheobase current (**D**), input resistance (**E**) and resting membrane potential (**F**) in sham + scramble (n = 15 cells of 4 mice), sham + shRNA (n = 12 cells of 3 mice), CFA + scramble (n = 9 cells of 3 mice), and CFA + shRNA (n = 15 cells of 4 mice) groups (*One-Way ANOVA* followed by *Tukey’s post-hoc* test. *F*_*(3,47)*_ = 3.012, *P* = 0.0393 (**D**); *F*_*(3,36)*_ = 5.689, *P* = 0.0027 (**E**); *F*_*(3,51)*_ = 0.825, *P* = 0.4861 (**F**). *P < 0.05, **P < 0.01)

Finally, we tested whether Rab11a affects the intrinsic property of neurons in lamina II of SDH. It’s shown that the average number of spikes, with incubation of scrambled-shRNA, was significantly increased in the CFA group compared with that of the sham group. Rab11a-shRNA incubation decreased the spike number in CFA group but not in sham group (Fig. 5A-B). In addition, CFA injection decreased the lowest current required to elicit an action potential (the rheobase current) and increased the input resistance, which were rescued by incubation of Rab11a-shRNA. However, Rab11a-shRNA did not affect the resting membrane potential in both CFA and sham groups (Fig. 5 C-E).

## Discussion

In the present study, we observe that increased expression of Rab11a in the SDH is significantly correlated with the development of inflammatory pain. Importantly, the mechanism underlying Rab11a’s effect in inflammatory pain process is likely related to its modulation of NMDAR-mediated EPSC and spiking of SDH neurons.

Consistent with our recent study [14] on a rat orofacial inflammatory pain model induced by CFA, we observed that approximately 89.15% of Rab11a-positive cells in the SDH of mice were positive for NeuN. Immunofluorescence and Western blotting analyses demonstrated a substantial increase in Rab11a expression in the SDH in mice with CFA injection, as quantified by both semi-quantitative and quantitative methods. Moreover, immunofluorescent results revealed a significant increase in the number of Rab11a-positive cells expressing the neuronal activation marker Fos at 3rd and 7th days post-CFA injection, which are consistent with previous reports [16–18]. Although the number of these cells was lower on the 7th day compared to the 3rd day, this difference did not reach statistical significant. Collectively, these findings provide compelling evidence that Rab11a may play a critical role in modulating inflammatory pain by affecting the neuronal function within the SDH.

To further investigate the impact of Rab11a on mechanical allodynia and neuronal excitability in the SDH, we utilized a Rab11a-specific down-regulated virus and administered it into the ipsilateral SDH. Intra-plantar injection of CFA rapidly induced mechanical allodynia on day 1, which persisted and worsened on the 3rd and 7th days. Rab11a-shRNA administration significantly increased the PWMT of the mice after CFA injection, further confirming the possible involvement of Rab11a in the process of inflammatory pain. Electrophysiologically, application of Rab11a-shRNA did not affect the presynaptic glutamate release and the postsynaptic AMPA receptor-mediated responses but reduced the NMDA receptor-mediated EPSCs and the number of spikes of neurons in lamina II of the spinal cord in mice with CFA injection. In our recent work in testing the Rab11a’s function in a CFA-induced orofacial inflammatory pain model in rats [14], we notice that Rab11a-shRNA similarly reduces the spike number of Sp5C neurons. However, Rab11a-shRNA also reversed the potentiated sEPSC frequency and amplitude [14]. Our recent work suggested that CFA activates the PI3K/AKT signaling pathway through up-regulating Rab11a expression, which can induce OFP hyperalgesia development furtherly, and Rab11a-shRNA downregulated these molecules’ expression and decreased the enhancement of Sp5C neuronal activity [14]. Previous study revealed that resveratrol could reverse the Aβ25-35-induced increase in the frequency of repetitive firing and the spike half-width of action potential via inhibiting the activation of PI3K/Akt signaling pathway [19]. Therefore, we speculate that Rab11a-shRNA considerably decrease the enhancement of spinal dorsal horn neuronal activity induced by CFA, which may through the inhibition of PI3K/AKT signaling pathway. Additionally, the inconsistency of sEPSC frequency and amplitude may be due to the differences in the animal species, pain model and nucleus being examined, which should be carefully explored in our future works.

Extensive researches have revealed the pivotal role of AMPA receptors in mediating rapid synaptic responses, while NMDA receptors undergo plastic changes during pathological pain rather than physiological conditions [20–22]. In the present study, we found the increased amplitude of NMDA current induced by CFA was completely abolished by Rab11a-shRNA treatment. Previous studies have indicated that Rab11a is involved in the enhancement of NMDAR function, by increasing the surface localization of recycling NMDARs [23], further suggesting the coordinate relationship between Rab11a and NMDAR.

Collectively, these results provide compelling evidence that increased Rab11a expression in the SDH contributes to inflammatory pain. The potential mechanism underlying this effect is Rab11a’s enhancement of NMDA receptor-mediated EPSC and spiking of SDH neurons. It is worth noting that further investigations are necessary to classify the specific cell types of Rab11a-positive neurons in the SDH. Additionally, delving into the mechanisms of synaptic plasticity (like NMDAR-mediated LTP) through which Rab11a influences the inflammatory pain process holds great promise for advancing our understanding of pain.

## Materials and methods

### Animals

A total of 81 wildtype C57BL/6J mice (male, 8 weeks old), provided by the Laboratory Animal Center of the Fourth Military Medical University [Xi’an, People’s Republic of China, license No. SCXK (Shan) 2019-001], were employed in the present study. All the animals could get free access to water and food and accommodated under a satisfactory environment [12-h light/dark cycle, controlled temperature (22 ± 1 ℃), and humidity (60 ± 5%)]. Most importantly, strict ethical guidelines for the pain investigation were needed, and the protocol (No. IACUC-20,211,101) was authorized by the local Ethics Committee on Animal Application for Research and Education of the Fourth Military Medical University.

This study can be divided into three parts. Firstly, we identified the location of Rab11a in the SDH. Then, chronic inflammatory pain model was constructed by intra-planar injection of CFA, and observed the changes of mechanical pain threshold, the number of Fos/Rab11a double-labeled cells and the expression level of Rab11a in the SDH at different time points after CFA injection. In the second part, Rab11a-specific interference virus (Rab11a-shRNA) was injected into the left SDH to observe the change of pain threshold of the ipsilateral hind paw. Combined with the first part, it was found that Rab11a positive cells were mainly NeuN positive cells, so the electrophysiological activity of these neurons in SDH after Rab11a knockdown was observed by the whole cell patch clamp technique. Thirdly, the possible mechanism of Rab11a involved in the development of chronic inflammatory pain was further explored by Immuno-Electron Microscopy and whole-cell patch clamp.

### Generation of inflammatory pain model

Chronic inflammatory pain model, has often been performed to investigate the potential mechanisms of peripheral pain disorders, was constructed as our previous studies [24, 25]. After anesthetized with 2% isoflurane, 50% CFA (10 µl, Sigma-Aldrich, Saint Louis, USA) was intra-plantar subcutaneously injected into the left hindpaw of mice. Meanwhile, 0.9% saline (10 µl) was used in the control animals.

### Von-Frey test

Pain withdrawal mechanical threshold (PWMT) of the left hind paw was tested via a range of Von-Frey filaments (Stoelting Company, Wood Dale, USA) as previous studies reported [26, 27]. Briefly, mice were placed in metal mesh grids (7 × 7 × 10 cm^3^) for 30 min before MPWT tests, which were applied at the same time point on baseline (days 0), days 1–7 after CFA/saline treatment. After mice adapted, an array of filaments (0.008, 0.02, 0.04, 0.16, 0.4, 0.6, 1, and 1.4 g) with increasing strengths (0.078, 0.196, 0.392, 1.568, 3.92, 5.88, 9.8, and 13.72 mN) were applied vertically investigate the mechanonociceptive threshold of the hind paw. The minimal bending force of filaments able to evoke 3 withdrawal response among 5 stimulations was considered the MPWT.

### Immunofluorescent histochemical staining

The mice were deeply anesthetized with 2% isoflurane and subsequently perfused transcardially with 25 ml of 0.01 M phosphate-buffered saline (PBS, pH 7.4), followed immediately by perfusion with 100 ml of a solution containing 4% (w/v) paraformaldehyde and 75% (v/v) saturated picric acid in 0.1 M phosphate buffer (PB, pH 7.4). The lumbar spinal cord was then rapidly removed and placed in 0.1 M PB containing 30% (w/v) sucrose overnight at 4℃. Subsequently, the spinal cord was cut into 30 μm thick serial sections using a freezing microtome (Kryostat 1720; Leitz, Mannheim, Germany). The sections were washed with 0.01 M PBS and immersed in PBS containing 0.3% Triton X-100 and 1% normal goat serum (NGS) for 30 min. All antibodies were diluted in PBS containing 5% (v/v) normal donkey serum (NDS), 0.3% (v/v) Triton X-100, 0.05% (w/v) sodium azide, and 0.25% (w/v) carrageenan (PBS-NDS, pH 7.4). For Rab11a/GFAP/Iba-1/NeuN immunofluorescent staining, the sections were incubated overnight at 4℃ with rabbit anti-Rab11a (1:200; 20229-1-AP, Proteintech, Chicago, USA), mouse anti-GFAP (1:4000; MAB3402, merckmillipore, Massachusetts, USA), mouse anti-NeuN (1:500; MAB377, merckmillipore), and goat anti-Iba-1 (1:200; ab5076, Abcam, Cambridge, UK). For Rab11a and FOS immunofluorescent staining, the sections were incubated with rabbit anti-Rab11a and mouse anti-FOS (1:500; ab11959, Abcam, Cambridge, UK) for 24 h at 4℃. After incubation with primary antibodies, the sections were washed and incubated with the appropriate fluorophore-conjugated secondary antibodies (1:200; Invitrogen, ThermoFisher, CA, USA) for 4 h at room temperature. Subsequently, the sections were washed with 0.01 M PBS three times for 10 min each. Finally, the sections were mounted on glass slides and observed using a laser scanning confocal microscope (FV1000, Olympus, Japan). The fluorescence intensity and number of double-labeled cells were measured using Image J software (NIH, Frederick, MD, USA), following the methodology described in our recent study [14].

### Western blotting

The mice were deeply anesthetized with 2% isoflurane and then perfused transcardially with 25 ml of pre-cooled 0.01 M PBS (pH 7.4). The left SDHs were promptly isolated and placed in centrifuge tubes on ice. Subsequently, the left SDHs were homogenized using a hand-held pestle in sodium dodecyl sulfate (SDS) sample buffer. The samples were heated at 100 ℃ for 10 min and loaded onto 10% SDS-polyacrylamide gels using standard Laemmli solutions (BioRad Laboratories, CA, USA). Electrophoresis was performed to separate the proteins, followed by electroblotting onto a polyvinylidene difluoride membrane (PVDF, Immobilon-P, Millipore, Hayward, CA, USA). The membranes were incubated in a blocking solution for 1 h and then gently agitated overnight with rabbit anti-Rab11a (1:200). The primary antibodies bound to the membrane were detected using a horseradish peroxidase (HRP)-conjugated anti-rabbit secondary antibody (1:5000; ZB-2301, ZSGB-BIO, Beijing, China). All reactions were visualized using the enhanced chemiluminescence (ECL) detection method. The densities of the protein bands were analyzed using Labworks Software (Ultra-Violet Products, UK).

### Injection of viral Vector

Mice were anesthetized with 2% isoflurane and secured on a stereotaxic apparatus (RWD Life Science, Shenzhen, China). Following established protocols [7], we performed the injection procedures. To downregulate Rab11a messenger RNA (mRNA) expression, we utilized Rab11a-specific small hairpin RNA (shRNA) encoded with adeno-associated viral (AAV) vectors (rAAV-CMV-DIO-(mCherry-U6)-shRNA (Rab11a)-WPRE-hGH polyA; BrainVTA, Wuhan, China) [15]. The AAV vectors containing Rab11a-shRNA were injected into the ipsilateral SDH after co-administration with the hSyn promoter virus carrying the cre enzyme (rAAV-hSyn-CRE-WPRE-hGH pA; BrainVTA). A similar injection procedure was followed for the administration of scramble shRNA as a control. In the second part of this study, the mice were randomly divided into four groups: Sham + scramble group, Sham + shRNA group, CFA + scramble group, and CFA + shRNA group. In the Sham + scramble group and CFA + scramble group, 0.2 µl of scramble shRNA virus was injected. In the Sham + shRNA group and CFA + shRNA group, 0.2 µl of Rab11a shRNA virus was injected ipsilaterally.

### Electrophysiological Recording

To investigate the neurophysiological properties of spinal dorsal horn (SDH) neurons, electrophysiological recordings were performed following our recent studies. On the 7th day after CFA or Sham operation, mice in the Sham + scramble group, Sham + shRNA group, CFA + scramble group, and CFA + shRNA group were anesthetized with 2% isoflurane. Lumbar spinal cord sections were then obtained and placed in oxygenated (95% O2 plus 5% CO2) pre-cooled artificial cerebrospinal fluid (ACSF) containing 248 mM sucrose instead of NaCl for 30 min at 4℃. Subsequently, 300 μm slices of the lumbar spinal cord were cut using a vibratome (Leica VT 1200 s, Heidelberger, Nussloch, Germany) and transferred into frozen oxygenated ACSF containing 124 mM NaCl, 1 mM NaH2PO4, 25 mM NaHCO3, 2 mM MgSO4·7H2O, 2.5 mM KCl, 25 mM glucose, 2 mM CaCl2, 3.0 mM pyruvate, and 1 mM ascorbate. The slices were allowed to recover at room temperature for 1 h before electrophysiological recording.

In voltage clamp mode, currents were recorded at a holding potential of -70 mV using recording pipettes filled with an intra-electrode solution consisting of 0.2 mM Tris-GTP, 0.4 mM EGTA, 4 mM Mg-ATP, 5 mM NaCl, 10 mM HEPES, 20 mM KCl, and 130 mM potassium gluconate (pH 7.2–7.4; osmolality 290–300 mOsm). Spontaneous excitatory postsynaptic currents (EPSCs) were recorded from layer I and layer II neurons using an Axon 700B amplifier (Molecular Devices Inc., CA, USA). AMPAR-mediated EPSCs were induced by repetitive stimulations at 0.02 Hz, and the neurons were voltage-clamped at -70 mV. For recording NMDA receptor-mediated EPSCs, local stimulations were delivered with a bipolar tungsten stimulating electrode connected to an isolation current stimulator [Natus Medical Incorporated (NASDAQ: PCLN-news, L6H5S1, Canada)] at an intensity of 20 µA. The recording pipettes were filled with a solution containing 102 mM cesium gluconate, 5 mM TEA chloride, 3.7 mM NaCl, 11 mM BAPTA [1,2-bis(2-aminophenoxy) ethane-N,N,N’,N’-tetraacetic acid], 0.2 mM EGTA, 20 mM Hepes, 2 mM MgATP, 0.3 mM NaGTP, and 5 mM QX-314 chloride (adjusted to pH 7.2 with CsOH, 280 to 300 mOsm). NMDA EPSCs were pharmacologically isolated in ACSF containing CNQX (6-cyano-7-nitroquinoxaline-2,3-dione; 20 mM). Neurons were voltage-clamped at + 30 mV, and NMDA EPSCs were evoked at 0.05 Hz. In current clamp mode, the firing patterns of SDH neurons in mice experiencing inflammatory pain (IP) and control mice were obtained by recording the trains of action potentials (APs) evoked by intracellular injection of depolarizing currents ranging from 0 to 200 pA (interval: 25 pA) for 400 ms.

### Statistical analysis

All data in the present study are presented as mean ± standard error of the mean (SEM). Statistical analysis and data visualization were performed using GraphPad Prism 9.1 software (GraphPad Software, Inc., La Jolla, CA, USA). ImageJ software was used to analyze the density of immunofluorescence (IF) and Western blotting (WB) bands. Statistical significance was assessed using One-Way ANOVA followed by Tukey’s post-hoc test and Two-Way ANOVA followed by Tukey’s post-hoc test. All experiments were conducted in a blinded manner to minimize bias.

## Data Availability

The data that support the findings of this study are available on reasonable request from the corresponding author.

## References

[1] Muley MM, Krustev E, McDougall JJ (2016). Preclinical Assessment of Inflammatory Pain. CNS Neurosci Ther.

[2] Conaghan PG, Cook AD, Hamilton JA, Tak PP (2019). Therapeutic options for targeting inflammatory osteoarthritis pain. Nat Rev Rheumatol.

[3] Todd AJ (2010). Neuronal circuitry for pain processing in the dorsal horn. Nat Rev Neurosci.

[4] Yu H, Cranfill SL, Luo W (2022). ErbB4(+) spinal cord dorsal horn neurons process heat pain. Neuron.

[5] Masuda T, Ozono Y, Mikuriya S, Kohro Y, Tozaki-Saitoh H, Iwatsuki K (2016). Dorsal horn neurons release extracellular ATP in a VNUT-dependent manner that underlies neuropathic pain. Nat Commun.

[6] Peirs C, Seal RP (2016). Neural circuits for pain: recent advances and current views. Science.

[7] Chen T, Taniguchi W, Chen QY, Tozaki-Saitoh H, Song Q, Liu RH (2018). Top-down descending facilitation of spinal sensory excitatory transmission from the anterior cingulate cortex. Nat Commun.

[8] Mignogna G, Fabrizi C, Correani V, Giorgi A, Maras B. Rab11A Depletion in Microglia-Derived Extracellular Vesicle Proteome upon Beta-Amyloid Treatment. Cell biochemistry and biophysics 2023. 10.1007/s12013-023-01133-4 PMC10257621 36995559

[9] Venugopal K, Chehade S, Werkmeister E, Barois N, Periz J, Lafont F (2020). Rab11A regulates dense granule transport and secretion during Toxoplasma gondii invasion of host cells and parasite replication. PLoS Pathog.

[10] Yu X, Taylor AMW, Nagai J, Golshani P, Evans CJ, Coppola G (2018). Reducing astrocyte calcium signaling in vivo alters Striatal Microcircuits and causes repetitive behavior. Neuron.

[11] Li D, Huang S, Zhu J, Hu T, Han Z, Zhang S (2019). Exosomes from MiR-21-5p-Increased neurons play a role in Neuroprotection by suppressing Rab11a-Mediated neuronal Autophagy in Vitro after traumatic brain Injury. Med Sci Monitor: Int Med J Experimental Clin Res.

[12] Udayar V, Buggia-Prévot V, Guerreiro RL, Siegel G, Rambabu N, Soohoo AL (2013). A paired RNAi and RabGAP overexpression screen identifies Rab11 as a regulator of β-amyloid production. Cell Rep.

[13] Xu P, Huang X, Niu W, Yu D, Zhou M, Wang H (2022). Metabotropic glutamate receptor 5 upregulation of γ-aminobutyric acid transporter 3 expression ameliorates cognitive impairment after traumatic brain injury in mice. Brain Res Bull.

[14] Liu M, Li X, Wang J, Ji Y, Gu J, Wei Y (2023). Identification and validation of Rab11a in rat orofacial inflammatory pain model induced by CFA. Neurochem Int.

[15] Monis WJ, Faundez V, Pazour GJ (2017). BLOC-1 is required for selective membrane protein trafficking from endosomes to primary cilia. J Cell Biol.

[16] Lee MJ, Jang M, Jung HS, Kim SH, Cho IH (2012). Ethyl pyruvate attenuates formalin-induced inflammatory nociception by inhibiting neuronal ERK phosphorylation. Mol Pain.

[17] Lin YR, Chen HH, Ko CH, Chan MH (2007). Effects of honokiol and magnolol on acute and inflammatory pain models in mice. Life Sci.

[18] Saeki A, Yamanaka H, Kobayashi K, Okubo M, Noguchi K (2022). Analgesic effect of gastrin-releasing peptide in the dorsal horn. Mol Pain.

[19] Yin H, Wang H, Zhang H, Gao N, Zhang T, Yang Z (2017). Resveratrol attenuates Aβ-Induced early hippocampal neuron excitability impairment via recovery of function of Potassium channels. Neurotox Res.

[20] Qiu S, Chen T, Koga K, Guo YY, Xu H, Song Q (2013). An increase in synaptic NMDA receptors in the insular cortex contributes to neuropathic pain. Sci Signal.

[21] Zhuo M (2009). Plasticity of NMDA receptor NR2B subunit in memory and chronic pain. Mol Brain.

[22] Bliss TV, Collingridge GL, Kaang BK, Zhuo M (2016). Synaptic plasticity in the anterior cingulate cortex in acute and chronic pain. Nat Rev Neurosci.

[23] Wang J, Lv X, Wu Y, Xu T, Jiao M, Yang R (2018). Postsynaptic RIM1 modulates synaptic function by facilitating membrane delivery of recycling NMDARs in hippocampal neurons. Nat Commun.

[24] Li YJ, Zhang K, Sun T, Wang J, Guo YY, Yang L (2019). Epigenetic suppression of liver X receptor β in anterior cingulate cortex by HDAC5 drives CFA-induced chronic inflammatory pain. J Neuroinflamm.

[25] Sun T, Wang J, Li X, Li YJ, Feng D, Shi WL (2016). Gastrodin relieved complete Freund’s adjuvant-induced spontaneous pain by inhibiting inflammatory response. Int Immunopharmacol.

[26] Zhang MM, Geng AQ, Chen K, Wang J, Wang P, Qiu XT (2022). Glutamatergic synapses from the insular cortex to the basolateral amygdala encode observational pain. Neuron.

[27] Zhu DY, Cao TT, Fan HW, Zhang MZ, Duan HK, Li J (2022). The increased in vivo firing of pyramidal cells but not interneurons in the anterior cingulate cortex after neuropathic pain. Mol Brain.

